# Serum IL-1RA levels increase from follicular to luteal phase of the ovarian cycle: A pilot study on human female immune responses

**DOI:** 10.1371/journal.pone.0238520

**Published:** 2020-09-03

**Authors:** Matthew Vetrano, Adam Wegman, Bryan Koes, Saurabh Mehta, Christine A. King

**Affiliations:** 1 Department of Microbiology and Immunology, SUNY Upstate Medical University, Syracuse, NY, United States of America; 2 Division of Nutritional Sciences, Cornell University, Ithaca, NY, United States of America; Dasman Diabetes Institute, KUWAIT

## Abstract

The immune responses exhibited by females are distinct from those of males. Females are known to generate, among others, higher levels of antibodies, greater interferon responses, and increased levels of inflammatory mediators in response to pathogens. Mounting evidence suggests that gonadal hormones play a key role in these differences. To better understand the effect of cycling hormones on the immune response, we sought to investigate the relationship between gonadal hormone fluctuations during the ovarian cycle and the levels of interleukin 1β and IL-1RA, both in circulation and in PBMCs in response to TLR4 stimulation, in healthy premenopausal females. To do this we measured the gonadal hormones 17β-estradiol, progesterone, and luteinizing hormone, and the cytokines IL-1β and IL-1RA in nine cycling females at several time points throughout one complete cycle. We evaluated 35 follicular, 17 ovulatory, and 44 luteal time points in our cohort and found a clear increase in serum levels of anti-inflammatory IL-1RA in the luteal phase, as compared to the follicular phase, and a positive correlation between both 17β-estradiol and progesterone and IL-RA. There was no difference in the serum levels of IL-1β and no difference in IL-1 β or IL-1RA produced in response to LPS by PBMCs isolated from different phases. Division of the cycle into sub-phases revealed an increase in the level of IL-1RA by ovulation that persisted through the luteal phase. These data suggest that significant changes in the immune response occur throughout the ovarian cycle in healthy females.

## Introduction

Human females are known to mount a more efficient immune response to vaccination, infection, and injury than males. This is in part due to a more Th2-predominant response in females, which is responsible for increased neutralizing antibody production [1]. For example, females can achieve similar antibody titers as males following inoculation with only half the standard dose of trivalent influenza vaccine [2]. These differences are thought to be a result of several factors, including sex-linked overexpression of the X-linked Toll-like receptor 7 gene in females relative to males, and the influence of gonadal steroid hormones on immune cell function and gene expression [3, 4].

Of these factors, gonadal hormones are of particular interest for several reasons. It has been speculated for some time that the sexual dimorphism in the immune response is due to differences in the sex steroid environment between males and females [5]. A substantial literature has developed underscoring this point. The human sex hormones estrogen and progesterone have been shown to have a broad range of both pro- and anti-inflammatory effects [6]. Estrogen and 17β estradiol binds the estrogen receptor expressed on both innate and adaptive immune cells. Estrogen signaling regulates neutrophil numbers and function [7], as well as macrophage and dendritic cell function modulating expression of inflammatory cytokines IL-6 and CCL2 [8–11]. Furthermore, estrogen has been shown to modulate adaptive cells of the immune system including Th1, Th2, Th17 and Tregs CD4+ T cell subsets, and CD8+ T cells, enhancing inflammatory gene expression and skewing the immune system to a TH2 response [12–14]. In males, testosterone levels are known to affect several immune cell populations in humans, a topic thoroughly reviewed in [15]. Androgens have been shown to promote the differentiation of neutrophils, mice lacking androgen receptors are neutropenic and show compromised host defense [16] while human females with increased level sof androgens due to polycystic ovarian syndrome exhibit neutrophilia [17]. In other studies, gonadectomized male mice exhibit increased TLR4 macrophage expression that results in elevated pro-inflammatory responses during infection suggesting that limiting myeloid cell responsiveness to pathogens is one possible mechanism by which androgens are immunosuppressive [18]. Subsequent studies demonstrated higher TLR4 expression, increased phagocytosis, and enhanced oxidative burst in female macrophages as compared to male macrophages [19] and a specific downregulation of TLR4 expression by testosterone *in vitro* [18]. IL-4 knockout C56BL/6 mouse bone marrow-derived DCs exposed to dihydroxy testosterone during antigen uptake activate T cells less effectively than unexposed DCs [20]. In other studies, low testosterone levels have been associated with higher B cell counts [21], whereas higher testosterone levels are associated with less potent antibody production following influenza vaccination [22]. Castrated mice show greater T cell proliferation after TCR stimulation [23], and administration of testosterone likewise reduces T cell proliferation in response to the OVA antigen [24]. All of these data point to a substantial role of sex steroids in the sex bias of the immune response; moreover, the dynamics of these hormones' fluctuation over the course of the ovarian cycle introduces another layer of complexity, which has yet been incompletely addressed.

The menstrual cycle comprises two major phases, the follicular and luteal phase. The first day of menstrual bleeding defines day 1 of the cycle and marks the beginning of the follicular phase, which continues until ovulation. Follicular phase typically persists for 12 to 16 days and is characterized by the presence of maturing ovarian follicles. During this phase, daily production of 17β-estradiol (E2) is approximately 36 μg/day, while progesterone (P) is very low, at 1 μg/day [25]. Towards the end of the follicular phase, a surge in luteinizing hormone (LH) occurs and is maintained for approximately 24–48 hours. The LH surge leads to increased intrafollicular proteolytic enzymes, destroying the basement membrane of the follicle and allowing for follicular rupture [26]. After follicular rupture, LH stimulates an increase in P and E2 levels, beginning the luteal phase and preparing the corpus luteum and endometrium for possible implantation by a zygote [27]. The second phase typically lasts from day 14 to day 28, and is characterized by increased E2 and P levels, with daily production at approximately 250 ug/day (~7-fold increase from follicular) and 25 ug/day (25-fold increase from follicular), respectively [25, 27].

We have little understanding of the changes in the immune response that occur in healthy cycling females over the course of the menstrual cycle. Increased plasma concentrations of the Th2 cytokine IL-4 have been reported in the luteal phase as compared to the follicular phase [28, 29], with Fass' group suggesting a possible role of increased E2 and P in promoting this shift. This is consistent with the notion of females’ greater tendency towards a Th2 response as compared to males being partly dependent on gonadal hormones. The same lab later reported an increased sensitivity of monocytes to endotoxin during the luteal phase [30], as well as an increase in the percentage of monocytes producing IL-1β. These latter two observations underscore both the relevance of our *ex vivo* stimulations with LPS, as well as our focus on the IL-1 family of cytokines.

The IL-1 cytokine family comprises 11 members, all of which promote the activity of cells of the innate immune system [31, 32]. Though the family has since been expanded, IL-1β was among the first described; it functions as a potent pyrogen, lymphocyte activator, and mediator of autoinflammation [33, 34]. Due to both the potency and extensive function of IL-1β, it must be tightly regulated; IL-1RA, its cognate antagonist, binds the target receptor of IL-1β, blocking further binding of the cytokine [31]. Current data suggest that the ratio of IL-1β and. IL-1RA concentration markedly affects the severity of some diseases [35–37]. Children with a disorder caused by mutations of IL1RN, the gene encoding IL-1RA, experience disorders including neonatal onset of sterile multifocal osteomyelitis, periostitis, and pustulosis [38]. After receiving FDA approved anakinra, a recombinant form of IL-1RA, children with IL1RN mutations experienced clinical remission with both peripheral blood counts and acute-phase reactant levels normalizing [38]. The pathogenic potential of IL-1β, and the reciprocal importance of IL-1RA was demonstrated in *in vivo* studies using IL-1RA deficient mice whereby chemical carcinogen exposure led to the development of more aggressive tumors and more potent angiogenic responses when compared to WT mice [39]. Together, IL-1β and IL-1RA both have substantial clinical relevance: the anti-IL-1β antibody canakinumab, along with anakinra, are FDA approved for a range of pathologies involving dysregulated inflammation.

Despite their importance, the dynamics of these cytokines over the course of the menstrual cycle has not been well-defined. Human urine samples taken over the course of the menstrual cycle show lowest levels of IL-1RA during menses; and experiments with LPS-stimulated monocytes show increased secretion of IL-1β following ovulation, along with an upward trend of IL-1RA that did not reach statistical significance [40, 41]. Later transcriptomics work showed enrichment of pro-inflammatory genes among up-regulated genes in the luteal phase as compared to the follicular phase following aerobic exercise, while IL-1RA gene expression was downregulated over the same interval [42]. These studies are limited by their use of indirect measurements of the cytokines in question and potentially confounding experimental conditions, and leave unknown the dynamics of the serum cytokine concentrations over the course of the normal menstrual cycle.

We hypothesized that the changes in the levels of E2 and P from the follicular to the luteal phase would influence the cellular activation response and the circulating levels of key IL-1 inflammatory cytokines. To investigate this effect over the course of the menstrual cycle, we analyzed the levels of IL-1β and IL-1RA during different phases throughout the cycle, in serum as well as *de novo* production by PBMCs, either non-stimulated or stimulated with LPS. Here we provide evidence for a robust increase in serum IL-1RA levels from the follicular to the luteal phase of the menstrual cycle. By dividing the cycle into sub-phases, we were able to more accurately elucidate the trends seen over the ovarian cycle [41]. Analysis of these 5 sub-phases—early and late follicular, ovulation, and early and late luteal—demonstrated the increase occurred by ovulation and was maintained throughout the luteal phase.

## Materials and methods

This study was a retrospective cohort analysis of a previous prospective study [43] utilizing de-identified subject data collected from university volunteers. The study was approved by the SUNY Upstate Medical University Institutional Review board (IRB-157178-1). The subject cohort included nine volunteers who were healthy, Caucasoid women between the ages of 19–29 years, not pregnant or taking oral contraceptives, with stable menstrual cycles (appr. 28 days), no history of medical problems, and normal BMI. Subjects were required to monitor symptoms experienced over three menstrual cycles and record their ovulation time while having blood drawn 9–12 times across a single cycle for cytokine and hormone analysis. Written informed consent was obtained from all volunteers.

The menstrual cycle diary consisted of a single page, self-report questionnaire to monitor symptoms [44]. The diary measured the magnitude of severity of 21 symptoms on a scale from 1–3. Briefly, if the volunteer did not experience any symptoms, the space corresponding to that symptom was left blank; if the symptom was mild (noticeable but not troublesome), the number 1 was recorded; if the symptom was moderate (interferes with normal activity), a 2 was recorded; and if the symptom was severe (temporarily incapacitating), a 3 was recorded.

The OvuQuick Ovulation predictor kit (Pharmasience Inc., Montreal) detects the presence or absence of LH in urine. Urine was collected between 10:00 and 20:00 and was not the first urine of the day. Date, cycle day and time were recorded when the sample was obtained to determine when ovulation occurred. The test was carried out by the volunteer according to the manufacturer’s instruction.

Venous blood samples (3 ACD; 2 clot) were obtained by venipuncture from the subjects three times a week for five weeks. Both serum and peripheral blood mononuclear cells (PBMC) were collected. The serum-separating tubes were left at room temperature to clot for 1h, then centrifuged at 2000 rpm for 20 minutes. Serum was removed, aliquoted and frozen at -70°C for future analysis. Peripheral blood mononuclear cells (PBMC) were isolated from fresh blood using density gradient centrifugation in Lymphoprep TM (Cederlane, Ontario).

PBMCs were cultured at 5 x 10^6^ cells/mL in RPMI-1640 supplemented with 5% FCS, 2mM L-glutamine, 1mM sodium pyruvate, and 50IU/mL penicillin-streptomycin. PBMCs were either left unstimulated or stimulated with the bacterial TLR4 ligand lipolysaccaride (LPS, 8μg/mL, Sigma) for 24h at 37°C in a humidified 5% CO_2_-incubator. Cell free supernatants were harvested at 24 h post-stimulation, aliquoted, and stored at -70°C for analysis.

The cytokines in this study were all measured by commercial ELISA assay. Samples were run in duplicate and all the samples collected for one volunteer during the menstrual cycle were run on the same plate. The lower limit of detection of the ELISAs were, for IL-1β ultrasensitive (0.083pg/mL, Biosource), for IL-1β (1.0pg/mL, Biosource), and for IL-1RA (22.0pg/mL, R&D Systems). ELISAs were carried out according to the manufacturer’s instruction.

E_2_, P, and LH (Immunocorp) were measured in serum samples by RIA according to the manufacturer’s instructions. All samples were run in duplicate and all samples collected for one volunteer were run at the same time. Sensitivities of the assays were 5pg/mL, 0.1ng/mL, and 0.46U/L, respectively.

### Statistical analysis

Cycle phases were defined on a per-subject basis. If multiple measurements were taken within a phase as defined, the median of those measurements was taken as the representative value of the phase or sub-phase. For repeated-measures analysis, one subject with missing values at one sub-phase was excluded. All statistical analyses were performed in GraphPad Prism (version 8.2.0 for OSX, GraphPad Software, La Jolla California USA, www.graphpad.com).

Statistical comparison of cycle timepoints was performed as follows. Where appropriate, unspecified cytokine measurements were imputed; these values were entered as the lower limit of detection for the assay in question. Data were then log (10) transformed, and tested for normality per cycle phase with the Shapiro-Wilk normality test [45]. The threshold for data to meet normality assumptions was prespecified as p > α = 0.05. Follicular and luteal phase mediator levels meeting normality assumptions were compared with the paired Student’s t-test; those not meeting normality assumptions were compared with the Wilcoxon signed rank test. The threshold for statistical significance of the pairwise tests was prespecified as p ≤ α = 0.05, and the Bonferroni correction was applied to correct for multiple comparisons.

For early/late follicular/luteal analysis, if all sub-phases of a given mediator met normality assumptions, a repeated-measures one-way ANOVA was performed. The Dunnett post-hoc test was performed on statistically significant ANOVAs, comparing the early and late follicular and luteal sub-phase levels to mediator levels at ovulation. If one or more sub-phases of a given mediator did not meet normality assumptions, a Friedman test was performed. Dunn’s post-hoc test was performed on statistically significant Friedman tests, comparing the early and late follicular and luteal sub-phase levels to mediator levels at ovulation. The threshold for statistical significance of ANOVAs, Friedman tests, and post-hoc tests was prespecified as p ≤ α = 0.05.

To test correlations between gonadal hormones and cytokine values, data were pooled from all patients at all five sub-phases, and subjected to a Spearman rank correlation test.

## Results

### Characteristics of the study population

**Fig 1:**
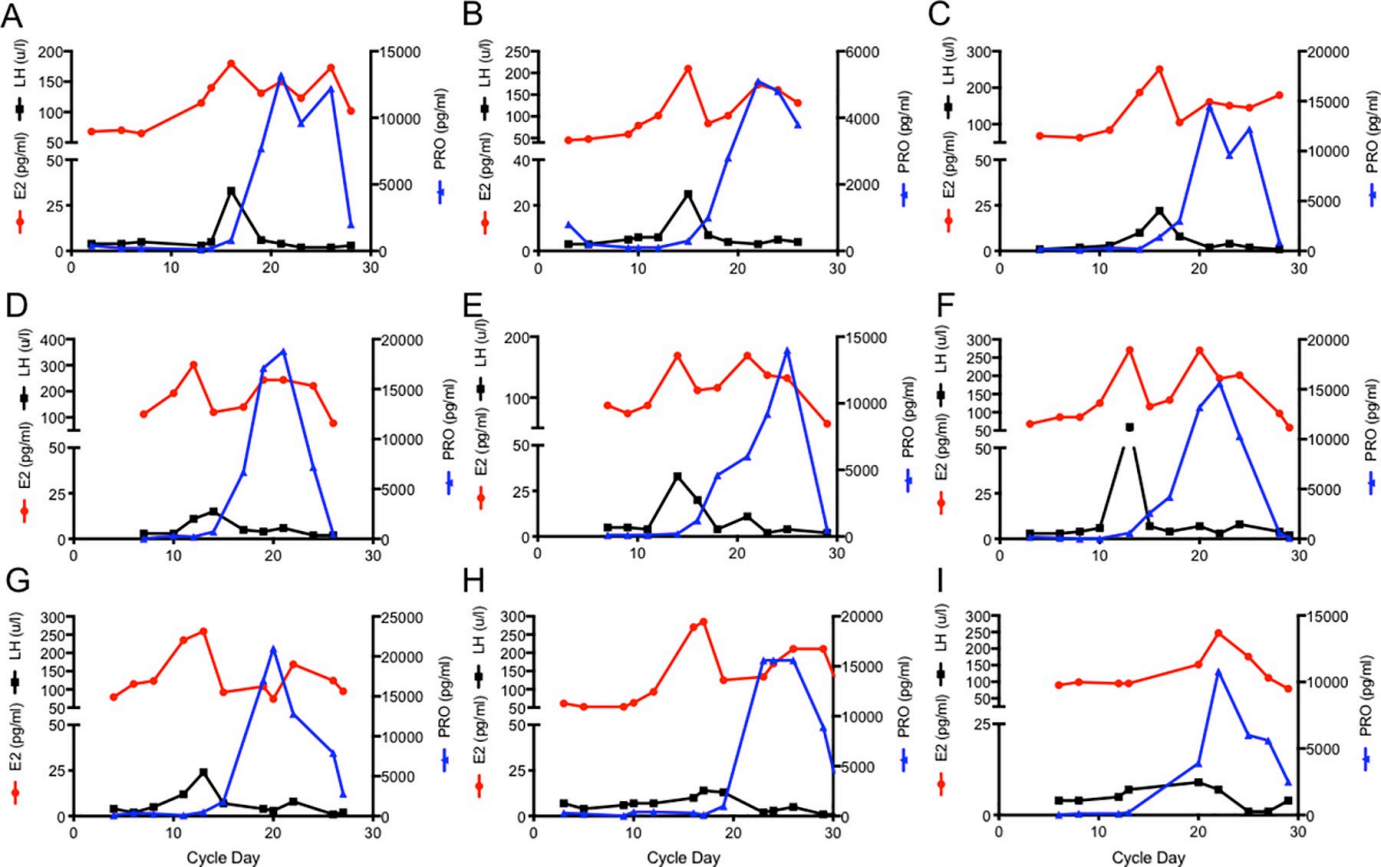
Subjects demonstrate stable menstrual cycles. Serum levels of E_2_, P and luteinizing hormone were measured by RIA in 9 female volunteers over one complete menstrual cycle.

A total of 9 healthy premenstrual cycling females were included in this study and their characteristics can be found in Table 1. Median (IQR) age was 23 years (20–25), cycle length was 29 days (28–29), and ovulation occurred on day 16 (14–16). Between 9 and 13 time points were taken for each participant over the course of 5 weeks and serum hormone levels were measured by RIA to define the phases of the cycle (Fig 1). E_2_ levels, median and (IQR), were 87.0pg/mL (65.0–102.0) and 138.5pg/mL (105.8–175.3), P was 0.1 pg/mL (0.1–0.2) and 7.8pg/mL (3.2–13.2), and LH was 4.0U/L (3.0–6.0) and 4.0U/L (2.0–5.0), in the follicular and luteal phase, respectively. LH levels during ovulation were 16.0U/L (13–26.5). Hormone analysis indicated that the nine volunteers had normal ovulatory cycles, with levels of E2, LH, and P within normal limits. In addition, mean symptom severity values derived from the menstrual diary during inter-menstrual and luteal phase were 1.86 (0.86–2.14) and 1.29 (0.93–2.64), respectively (n = 7) and indicated no significant premenstrual syndrome symptoms [44].

**Table 1:** Characteristics of the study participants.

Characteristic	Participants (n = 9)	
**Age in years**	23 (20–25)*	
**Cycle Length in days**	29 (28–29)	
**Ovulation Day**	16 (14–17)	
**HgB, g/L**	138.0 (135.0–143.0)	
**WBC, 10**^**9**^ **cell/L**	5.7 (5.3–5.7)	
**Monocytes, 10**^**9**^ **cell/L**	0.4 (0.4–0.5)	
**Lymphocytes, 10**^**9**^ **cell/L**	1.6 (1.6–1.7)	
**Neutrophils, 10**^**9**^ **cell/L**	3.4 (2.6–3.5)	
	**Follicular (n = 35)**	**Luteal (n = 44)**
**17beta-Estradiol, pg/mL**	87.0 (65.0–102.0)	138.5 (105.8–175.3)
	*15*.*0–350*.*0*^*†*^	*15*.*0–350*.*0*^*†*^
**Progesterone, ng/mL**	0.1 (0.1–0.2)	7.8 (3.2–13.2)
	*0*.*1–0*.*7*^*†*^	*2*.*0–25*.*0*^*†*^
**Luteinizing Hormone, u/L**	4.0 (3.0–6.0)	4.0 (2.0–5.0)
	*1*.*9–12*.*5*^*†*^	*0*.*5–16*.*9*^*†*^

*median, (interquartile range)

^†^expected range

### Heightened anti-inflammatory IL-1RA levels during the luteal phase of the menstrual cycle

**Fig 2:**
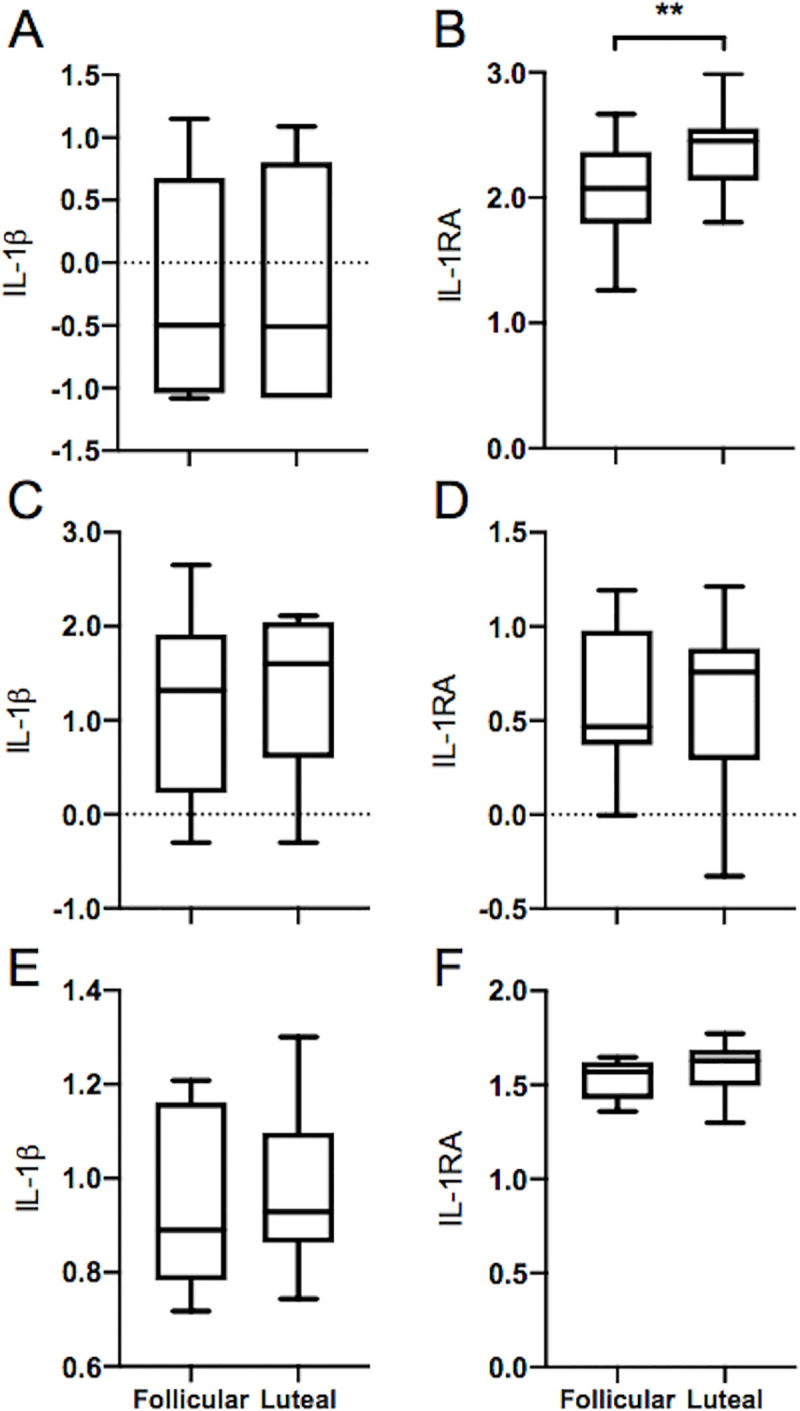
Serum IL-1RA levels increase from the follicular to luteal phase. Comparison of mediator levels between follicular and luteal phase of the menstrual cycle. Shown are median levels of IL-1β and IL-1RA from: serum (A, B); naïve, unstimulated PBMCs (C, D); and LPS-stimulated PBMCs (E, F). Data were log-transformed before analysis to meet normality assumptions; whiskers denote 5th and 95th percentiles, ** denotes p ≤ 0.005.

Females tend to have more robust immune responses than males and some of these differences are thought to be due to gonadal hormones influencing the outcome of the immune response. To investigate any changes in inflammatory cytokine levels over the menstrual cycle we analyzed serum levels of potent pro-inflammatory IL-1β and anti-inflammatory IL-1RA levels at each time point by ELISA. We found levels of IL-1RA were readily detectable in serum, while IL-1β levels were 100x lower and only detectable with an ultrasensitive ELISA. To determine if any differences occurred between the phases, we divided each participant’s cycle into follicular and luteal phases based on individual hormone levels. Follicular phase was defined as the interval following day 1 of menses to ovulation, exclusive. Luteal phase was defined as the interval following ovulation to day 1 of menses, exclusive. Our data showed that serum levels of IL-1RA significantly increased from the follicular to the luteal phase; log transformed median (IQR) values were 2.07 (1.79–2.37) vs. 2.45 (2.14–2.56), p = 0.004, (Fig 2B), while serum IL-1β levels were not different between the two phases, -0.50 (-1.04–0.68) vs -0.51 (-1.08–0.80), p = 0.83 (Fig 2A).

To evaluate any differences in the ability of peripheral blood mononuclear cells to respond to the common bacterial ligand, LPS, PBMCs isolated from peripheral blood at several time points over the menstrual cycle were stimulated with or without LPS *in vitro*. At 24 h, cell-free supernatants were harvested and IL-1β and IL-RA levels were assessed by ELISA. Our analysis, reported as median (IQR), found no differences in the levels of IL-1β (Fig 2C) or IL-1RA (Fig 2D) produced in the follicular vs the luteal phase in unstimulated cultures; 1.32 (0.23–1.92) vs. 1.60 (0.60–2.05), p = 0.60, and 0.47 (0.37–0.98) vs. 0.76 (0.29–0.88), p = 0.96, respectively; and no significant differences in TLR4-LPS stimulated cultures, IL-1β (Fig 2E) and IL-1RA (Fig 2F), 1.19 (0.90–3.84) vs. 1.09 (0.91–3.87), p = 0.73, and 1.57 (1.42–1.62) vs. 1.63 (1.50–1.69), p = 0.38, respectively.

### Anti-inflammatory associated serum IL-1RA levels are increased by ovulation

**Fig 3:**
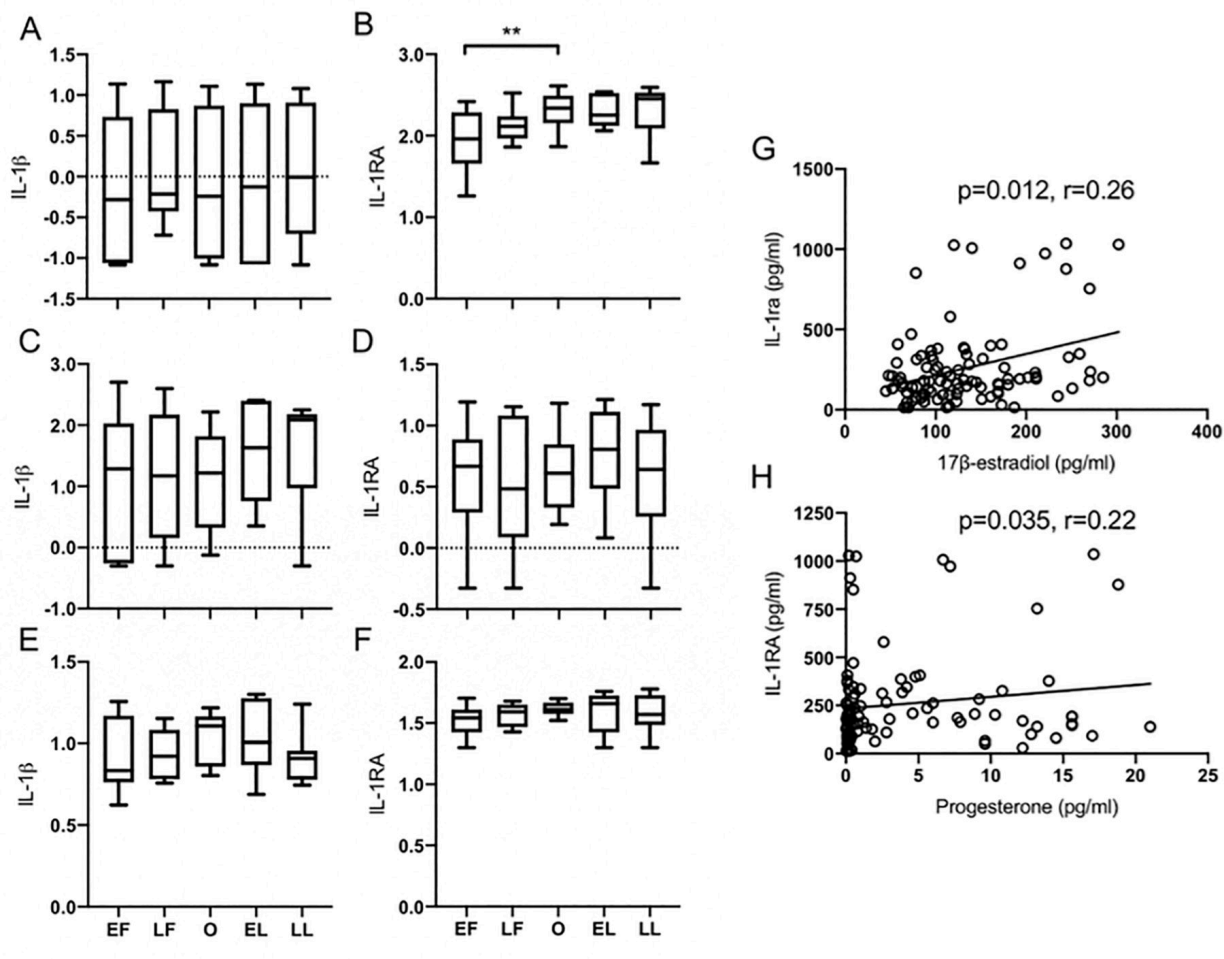
Serum IL-1RA levels increase over the cycle while being maintained in the luteal phase and are positively correlated with E_2_ and P. Patient samples were grouped into 5 distinct sub-phases of the menstrual cycle. Shown are median levels of IL-1β and IL-1RA from: serum (A, B); naïve unstimulated PBMCs (C, D); and LPS-stimulated PBMCs (E, F). Serum IL-1RA positively correlated with gonadal hormones E_2_ (G) and P (H); values from all five sub-phases were pooled from all patients. Data were log-transformed before analysis to meet normality assumptions; whiskers denote 5th and 95th percentiles; ** denotes p ≤ 0.005.

Our data suggest an increase in the levels of circulating IL-1RA during the luteal phase of the menstrual cycle. To begin to understand when these differences emerge during the cycle and how long they were maintained, we divided the individual phases into 5 distinct sub-phases, early/late follicular (EF/LF), early/late luteal (EL/LL) and ovulation (O) [41]. Early/late follicular and early/late luteal sub-phases were defined as equal intervals on either side of the midpoint of the entire follicular and luteal phases, respectively, and ovulation was defined by LH peak. Analysis of serum revealed significantly increasing IL-1RA levels (Fig 3B) from early follicular to ovulation, represented as median (IQR) and determined to be 1.96 (1.66–2.8) vs. 2.34 (2.15–2.49), p<0.01, with no significant changes in IL-1β observed (Fig 3A), p = 0.47. The increased levels of IL-RA within the cycle were evident at ovulation and persisted over the EL and LL phases. We observed no differences in IL-1β or IL-1RA production by PBMCs isolated at different sub-phases in either TLR-LPS stimulated (Fig 2E and 2F) or non-stimulated cultures (Fig 2C and 2D).

### Serum IL-1RA levels positively correlate with gonadal hormone levels

To determine if there was any relationship between serum hormone and cytokine levels, we carried out Spearman correlation tests to evaluate the relationship between gonadal hormones and cytokines. Our data showed a weak but positive correlation between IL-1RA and E2 (r = 0.26, p = 0.01) and between P and IL-1RA (r = 0.22, p = 0.04) (Fig 3G and 3H, respectively), suggesting that increased estradiol and progesterone was associated with a rise in IL-1RA levels.

## Discussion

Females are known to have enhanced immune responses when compared to males. In principle, the fluctuations in gonadal steroid hormones during the menstrual cycle have the potential to both explain part of these differences, as well as adjust the immune milieu more generally. However, the dynamics of major immune cytokines in serum over the course of the cycle are not fully understood. Here, we provide evidence for an increase in serum IL-1RA by ovulation that persists through the luteal phase in young, eumenorrheic women.

The IL-1 family of cytokines and their receptors play a central role in the modulation of innate immunity and inflammation [46]. We investigated IL-1β, a pro-inflammatory positive effector of the IL-1 system, and IL-1RA, an anti-inflammatory negative regulator of the IL-1 system. Previous investigations into IL-1RA production have shown that levels were lowest during menses in urine samples [41]. In another report, secreted IL-1RA trended upwards (though did not reach statistical significance) following ovulation, while levels of cell-associated IL-1RA were significantly increased post-ovulation in monocytes [40]. These observations are broadly consistent with our data. Regarding potential origins of this increase from the follicular to luteal phase, it should be noted that cytokine secretion is not confined to the vascular compartment. Along with circulating monocytes, epithelial cells of the endometrium have been implicated: a significant increase in secretion of IL-1RA from endometrial cells is observed from the follicular to luteal phase [47]. Taken together, our findings and published data suggest a shift in the cytokine environment at ovulation and through the end of the cycle.

Given the above, it is important to consider the possible drivers of this shift. As discussed, there is a strong biological rationale that cycling gonadal hormones themselves affect the immune response in women. Sex steroids play a major role in the sex bias of the immune response and have been shown to exert specific effects on female immunocompetence at the cellular and molecular level [48]. E2 receptors (ERs) are expressed on a majority of the innate and adaptive immune system cells, including T cells, B cells, neutrophils, macrophages, dendritic cells, and natural killer cells [49]. Transition into menopause, characterized by declining E2 levels, increases susceptibility to infection while decreasing vaccine efficacy. Post-menopausal women also show upregulation of TNF, IL-1β, IL-10, and IL-6, while showing diminished phagocytic capacity of DCs, leading to impaired antigen presentation and activation of the adaptive immune response. Moreover, E2 has been shown to block inflammatory effects caused by IL-1β and LPS in human and rat uteruses [50]. We found a modest but statistically significant correlation between both P and E2 levels and IL-1RA levels in our cohort when analyzed by sub-phase, consistent with prior observations of dampened IL-1β effects in the presence of E2.

Future studies should further investigate the clinical significance of a post-ovulation anti-inflammatory shift. A 2006 review of the relevant literature suggested that pre-menopausal breast cancer patients who underwent resection during the luteal phase had higher disease-free survival (DFS) rates than patients who underwent resection at other cycle times, despite some equivocal evidence [51]. More recently, a systematic review and meta-analysis found the literature on the matter to be inconclusive, but noted that the studies supporting the benefits of surgery in the luteal phase were generally more robust, with larger cohorts and longer follow-up times [52]. One such prospective study reported decreased DFS when surgeries were scheduled in the follicular phase of the menstrual cycle [53]. This effect was independent of the hormone receptor status of the tumors, indicating that the effect was not due to an intrinsic susceptibility of the tumors themselves to fluctuating hormone levels. Rather, the authors note that the immunosuppressive effect of adrenergic stimulation (e.g., from perioperative anesthesia) is exaggerated during the follicular phase in animal studies, possibly allowing for escape of tumor cells released during the resection. This suggests a potential mechanistic link between cycle phase, immune surveillance, and post-operative DFS. While the full mechanism for the increased survival rate is unclear, our data suggest that circulating anti-inflammatory cytokines may be associated with better clinical outcomes observed during luteal phase.

## Supporting information

S1 Table Individual serum and culture levels of cytokines over 5 sub-phases (previously defined on a per-subject basis) of the ovarian cycle. (DOCX)
