# Supplementary material for: Serum IL-1RA levels increase from follicular to luteal phase of the ovarian cycle: A pilot study on human female immune responses
Source: PLoS One. 2020 Sep 3;15(9):e0238520. doi: 10.1371/journal.pone.0238520 (PMC7470260; doi:10.1371/journal.pone.0238520)
Supplement: S1 Table — (DOCX) [file pone.0238520.s001.docx]

**S1 Table. Individual serum and culture levels of cytokines over 5 sub-phases (previously defined on a per-subject basis) of the ovarian cycle.**

|  | **EF** | **LF** | **O** | **EL** | **LL** |
| --- | --- | --- | --- | --- | --- |
| **IL-1β**^†^ **(pg/ml)** | 0.9 (0.09-6.0)^*^ | 0.6 (0.4-8.0) | 0.6 (0.1-8.3) | 0.8 (0.1-8.5) | 1.2 (0.2-8.6) |
| **IL-1RA**^†^ **(pg/ml)** | 92.2 (45.4-195.5) | 130.6 (92.5-173.9) | 218.9 (142.3-309.6) | 181.5 (132.2-334.1) | 286.1 (125.2-337.3) |
| **IL-1β**^‡^ **(pg/ml)** | 19.4 (0.6-134.0) | 20.6 (1.8-150.0) | 20.4 (2.5-70.5) | 46.1 (6.6-249.4) | 122.7 (10.4-151.0) |
| **IL-1RA**^‡^ **(ng/ml)** | 4.7 (2.0-7.7) | 3.1 (1.3-12.3) | 4.1 (2.2-7.1) | 6.4 (3.0-13.3) | 4.4 (2.0-9.5) |
| **IL-1β**^§^ **(ng/ml)** | 1903.9 (8.0-6275.9) | 2860.6 (10.0-7855.9) | 3183.3 (9.2-12118.7) | 1066.6 (9.7-11708.5) | 13.0 (7.7-5992.3) |
| **IL-1RA**^§^ **(ng/ml)** | 34.9 (26.6-40.9) | 39.2 (29.4-44.7) | 40.8 (37.7-46.5) | 45.7 (26.9-53.2) | 37.3 (30.6-53.9) |

^*^median (interquartile range), ^†^Serum, ^‡^PBMCs at rest, ^§^LPS-stimulated PBMCs
